# Assessing the Readability and Quality of Online Patient Information Regarding Aspirin-Exacerbated Respiratory Disease

**DOI:** 10.7759/cureus.117217

**Published:** 2026-09-30

**Authors:** Faiz A Shaikh, Huseyin Turacli, Rakesh Mistry, Raj Lakhani

**Affiliations:** 1 Otolaryngology, Epsom and St Helier University Hospitals NHS Trust, London, GBR; 2 Foundation Programme, Wexham Park Hospital, Slough, GBR; 3 Otolaryngology, St George’s University Hospitals NHS Foundation Trust, London, GBR

**Keywords:** aerd, aspirin-exacerbated respiratory disease, discern instrument, health literacy, online health information, patient education, readability, samter's triad

## Abstract

Background

The internet has revolutionised the way in which patients obtain information regarding their health. In order to be accessible, Health Education England recommend patient information should be at the reading age of an average 11-year-old. Effective management of aspirin-exacerbated respiratory disease (AERD) is complex and includes a combination of medical (including aspirin desensitisation and biological treatments) and surgical therapy to improve quality of life and reduce recurrence. We objectively assess the quality and readability of online patient materials on AERD.

Methods

The three search engines utilised were Google (Google LLC, Mountain View, CA, USA), Bing (Microsoft Corporation, Redmond, WA, USA) and Yahoo (Yahoo Inc., New York City, NY, USA). Three specific search terms (‘Samter’s Triad’, ‘ASA Triad’, and 'AERD') were used to identify the top 40 websites from each search result. After applying eligibility criteria, a total of 30 websites were finally analysed. Selected websites were assessed for readability using the Flesch-Kincaid Reading Ease Score (FRES), Flesch-Kincaid Grade Level (FKGL), and the Gunning-Fog Index (GFI). The quality of information available online was also assessed using the DISCERN tool.

Results

The average FRES was 36.95, which correlates to a US grade level of 11.5 (16-17-year-olds). The average GFI score was 15.07, which equals that of a 21-year-old. According to the DISCERN criteria, the overall quality of the health information was poor.

Conclusion

Health information available on the internet regarding AERD is overall of poor quality. The information available is written above Health Education England’s recommended reading level, which may reduce a patient’s understanding of AERD. As more patients turn to the internet to obtain health information, it is of utmost importance that information is validated and evidence-based to counter the spread of misinformation.

## Introduction

Aspirin-exacerbated respiratory disease (AERD) affects approximately one in 10 patients who suffer from asthma [1,2]. It is characterised by the worsening of bronchial asthma in response to the ingestion of aspirin or another non-steroidal anti-inflammatory drug (NSAID). The German-American immunologist Max Samter investigated this subset of patients and found that many had concomitant nasal polyps and rhinosinusitis [3]. The pathophysiology is driven by a profound dysregulation of the arachidonic acid cascade [4]. When COX-1 enzymes are inhibited by aspirin or NSAIDs, the metabolic pathway shunts toward the 5-lipoxygenase pathway, causing an overproduction of pro-inflammatory cysteinyl leukotrienes and a severe drop in anti-inflammatory prostaglandin E2 (PGE2) [5]. This subsequently causes increased vasodilatation and permeability of the nasal vasculature, resulting in mucosal oedema and increased mucus production, which manifests symptomatically as nasal congestion and rhinorrhoea.

AERD is considered a recalcitrant form of chronic rhinosinusitis (CRS) where early and accurate diagnosis is essential to initiate prompt multispecialty medical and surgical management to prevent irreversible airway remodelling, mitigate severe asthma exacerbations, and preserve the patient's quality of life [6]. Its management usually involves an allergist, a rhinologist, and/or a respiratory physician. It remains a significantly underdiagnosed condition; recent studies indicate that nearly a quarter (24.4%) of AERD patients experience a diagnostic delay of a year or more, which is directly associated with increased disease morbidity and higher systemic steroid use [7]. Diagnostic delay in AERD has been attributed to a lack of clinical or societal awareness of the condition and the condition’s slowly progressive nature, with many requiring multispecialty management [7]. This is further compounded by the time course of the disease, as most patients are diagnosed in adulthood, as the symptoms are slowly progressive [8]. In addition, many patients often do not associate their symptoms with aspirin because they already experience fluctuating baseline asthma and nasal congestion, making it difficult to distinguish a specific medication-induced reaction from a routine flare-up [9]. Diagnosis of AERD is often delayed, with some patients experiencing more than 10 years between symptom onset and correct diagnosis [10]. This delay results in patients utilising the internet in search of an explanation for their symptoms, thus posing new challenges for healthcare professionals who must reconcile patient expectations shaped by online resources with evidence-based clinical reality [11].

Health literacy is a term that has had over 250 recorded definitions in academic literature [12]. However, the most comprehensive and latest definition by Liu et al. in 2020 defines health literacy as ‘the ability of an individual to obtain and translate knowledge and information to maintain and improve health in a way that is appropriate to the individual and system contexts [13]. The National Literacy Trust reports that the average British adult has a reading age of that of an 11-year-old and estimates that 71 million Britons are ‘functionally illiterate', as they are classed as having very poor literacy skills [14].

The internet has revolutionised the way that patients interact with healthcare. It is vital in building health literacy [15]. In April 2021, the Office for National Statistics identified 92.5% of the population in the United Kingdom as regular internet users, which represents a 276% increase over the last 20 years [16]. This has been perpetuated by the COVID-19 pandemic, in which access to healthcare services has been limited. One recent study identified patients’ increasing reliance on Google searches for information on their health conditions since the COVID-19 pandemic started [16]. Whilst the internet provides a wealth of information available within seconds, ensuring the information is high quality, accurate and understandable for patients remains problematic [17]. 

Health literacy relies heavily on two pillars: the quality and the readability of available information. In this context, quality refers to the accuracy, reliability, and evidence-based nature of the content, ensuring it is transparent and free from commercial bias [18]. Readability describes the structural complexity of the text, typically measured by vocabulary difficulty and sentence length, which dictates the formal education level required to comprehend it. Both factors are fundamentally important to patient safety and empowerment. Poor quality information exposes patients to medical inaccuracies and potentially harmful treatments [19]. Conversely, if the readability of a resource exceeds the average patient's literacy level, even the highest quality clinical information becomes inaccessible, which has been directly linked to reduced uptake of healthcare services and poorer clinical outcomes [17]. Therefore, independent of the quality of health information sources, poor literacy often translates into poor decision-making, which can have lasting repercussions when exploring treatment options. In addition, poor literacy has also been linked to reduced uptake of healthcare services as well as increased admissions to hospital with a concomitant rise in mortality [20, 21]. This study aims to evaluate the readability and quality of online health information resources about AERD.

This article was previously presented as a poster at the Collaborative Care in ENT: Multidisciplinary Teams in Action event on May 1, 2026, at the Royal Society of Medicine in London, UK.

## Materials and methods

An internet search was completed in August 2021 using three of the most widely recognised search engines (Google (Google LLC, Mountain View, CA, USA), Bing (Microsoft Corporation, Redmond, WA, USA) and Yahoo (Yahoo Inc., New York City, NY, USA)) to identify text and image-based patient information resources for AERD. Three specific search terms (‘Samter’s Triad’, ‘ASA Triad’, and 'Aspirin-exacerbated respiratory disease (AERD)') were used to identify the top 40 websites from each search result, yielding a total of 360 initial results (3 search terms × 3 search engines × 40 websites). While search engine behaviour data indicates that up to 75% of users never scroll past the first page of results, evaluating the top 40 websites was chosen to ensure a comprehensive capture of the information landscape that a highly motivated patient might encounter. To ensure the search accurately captured the diverse terminology a patient might use or encounter, search terms were selected to encompass both the historical and contemporary clinical nomenclature for the condition. To capture a true reflection of what patients may search for, no additional advanced search settings were used. Websites were included if the intended audience was patients with AERD and the website was in English. In this study, we refer to the condition of AERD as a patient suffering from chronic rhinosinusitis, asthma, and nasal polyposis in addition to a sensitivity to aspirin. Websites that were excluded included blogs, forums and newspaper articles, as well as those with an intended target audience of healthcare professionals. Multimedia content, such as YouTube and social media platforms (e.g., Instagram, Facebook, Twitter, and TikTok), was excluded, as these platforms also rely on video content as their primary source of information. Any duplicate websites were excluded.

Any included websites were categorised by source. The categories selected were non-clinical health information sites, clinical providers (including both hospital and private clinics), professional associations, general information websites, a single patient-run website, and charitable organisations.

Readability was assessed using the Flesch Reading Ease (FRES), Flesch-Kincaid Grade Level (FKGL), and the Gunning-Fog Index (GFI) [22-24]. FKGL was included because it translates reading difficulty into an academic grade level, mapping directly to recommended patient reading ages. Simple Measure of Gobbledygook was excluded to avoid redundancy, as the GFI already fulfils a similar role by estimating required education years based on polysyllabic word density (Appendix A). The FRES score uses a mathematical formula based on the number of words and sentences to calculate a score out of 100 that correlates to the difficulty in reading (Appendix B). The FKGL score uses the same parameters as the FRES but instead utilises the United States of America’s grade Level to contextualise the numerical score. The GFI is a validated tool measuring the average sentence length and the number of trisyllabic (or more) words. From this, it generates a reading level used to determine the estimated number of years in formal education required to comprehend a passage of text in a single read. A summary of the scoring systems is in Table 1 [22-24].

**Table 1:** Summary of the Scoring Systems References: [22-25]

Scoring System	What It Measures	How It Works	Score Interpretation
Flesch Reading Ease Score	Readability	Analyses average sentence length and average syllables per word.	Scale of 0-100; Higher scores indicate easier reading (e.g., 90-100 is easily understood by an 11-year-old).
Flesch-Kincaid Grade Level	Readability	Uses sentence length and syllable count to calculate an equivalent US academic grade level.	Scores correlate directly to US school grades (e.g., a score of 8.0 is readable by an 8^th^ grader, or a 13–14-year-old).
Gunning-Fog Index	Readability	Measures sentence length and the percentage of complex, polysyllabic words (3+ syllables).	Estimates the years of formal education required to understand the text (e.g., a score of 15 equals a university level).
DISCERN Instrument	Quality and reliability	16-question tool using a 5-point Likert scale to assess reliability and treatment info.	Total score out of 75. (63-75 = Excellent; 51-62 = Good; 39-50 = Fair; 27-38 = Poor).

Mean, standard deviation and 95% confidence intervals were calculated for all readability scores. Readability scores were compared between source types using a one-way ANOVA test with statistical significance set at p < 0.05.

Quality was assessed using the validated DISCERN instrument [25]. This comprises 16 questions that are split into three categories, where each question is rated using a five-point Likert scale. The first section determines if the website publication is reliable, and the second assesses the quality of information on treatment choices. The third section (Question 16) requires the assessor to provide an intuitive overall rating of the publication based on the preceding sections. To objectively quantify and categorise the overall quality of the information for this study, we calculated the total sum of the first 15 questions. This yields a maximum possible score of 75 and a minimum of 15 (Appendix C). Those that are scored 63-75 are considered ‘excellent’, 51-62 are ‘good’, 39-50 are ' fair ', and finally 27-38 are ‘poor’. Two authors assessed the websites independently using the DISCERN instrument. The scores between the two authors were averaged, and inter-rater reliability was calculated using the interclass correlation coefficient (ICC). The coefficient is between 0 and 1, with scores below 0.2 indicating poor agreement between the assessors, whereas a score above 0.8 signifies excellent agreement. The DISCERN scores were compared between source types using a one-way ANOVA test with a statistically significant set at p < 0.05.

All statistical analyses were completed with IBM SPSS version 27 software for Mac OSX (IBM Corporation, Armonk, NY, USA). The distribution of the study variables was amenable to parametric analysis, and mean values were compared using the one-way analysis of variance (ANOVA). As this study exclusively analysed publicly available data and involved no human or animal subjects, formal ethics committee approval was not required. The Patient Society category contained only one website and was excluded from all between-source ANOVA comparisons. These analyses, therefore, included 29 websites across four source categories.

## Results

From the initial searches across all three search engines, a total of 360 websites were identified. Once the predefined exclusion criteria were applied (removing blogs, forums, non-English sites, and resources targeted at healthcare professionals) and all duplicates were excluded, this yielded a final total of 30 suitable, unique websites for analysis.

These source types are summarised in Table 2. These included 11 healthcare information sites, 14 clinical providers, including both hospital and private clinics, two professional associations, general information websites, and a single patient-run website. No charitable organisation websites were identified.

**Table 2:** Website Source Types

Source Type	Number (n = 30)	Percentage
Clinical Providers	14	46.7%
Healthcare Information Sites	11	36.7%
Professional Associations	2	6.7%
General Information Websites	2	6.7%
Patient-Run Website	1	3.3%
Charitable Organisations	0	0.0%

The mean readability scores and confidence intervals are summarised in Table 3.

**Table 3:** Mean Readability Scores and Confidence Intervals

Readability Scoring System	Mean	Standard Deviation	95% Confidence Interval
Flesch Reading Ease Score	36.95	11.77	32.56-41.35
Flesch-Kincaid Grade Level	11.52	2.03	10.76-12.28
Gunning-Fog Index	15.07	2.44	14.16-15.99

A comparison of readability scores by source type is presented in Table 4.

**Table 4:** Comparison of Readability Scores by Source Type Readability scores did not differ significantly between source categories for FRES (p = 0.47) or FKGL (p = 0.20). GFI scores differed significantly between source categories on the omnibus ANOVA (p = 0.04). Descriptive statistics include all 30 websites. ANOVA p-values were calculated using the four source categories containing more than one website (n = 29), excluding the Patient Society category (n = 1). n: number of websites; FRES: Flesch Reading Ease Score; FKGL: Flesch-Kincaid Grade Level; GFI: Gunning-Fog Index.

Readability Tool	Clinical Health Providers (n = 14)	Non-clinical Health Providers (n = 11)	Professional Associations (n = 2)	Encyclopaedia Website (n = 2)	Patient Society (n = 1)	Total (Average Across All Sources N = 30)	P-value
FRES	34.5	41.10	32.65	31.00	45.40	36.95	0.47
FKGL	12.01	10.63	13.05	12.60	9.20	11.52	0.20
GFI	15.94	13.78	17.98	14.93	11.61	15.07	0.04

A comparison of the overall quality of information, as assessed by DISCERN scores across source types, is shown in Table 5.

**Table 5:** Comparison of DISCERN Scores by Source Type DISCERN scores did not differ significantly between the four source categories included in the ANOVA (p = 0.07). Descriptive statistics include all 30 websites. ANOVA p-values were calculated using the four source categories containing more than one website (n = 29), excluding the Patient Society category (n = 1). n: number of websites

	Clinical Health Providers (n = 14)	Non-clinical Health Providers (n = 11)	Professional Associations (n = 2)	Encyclopaedia Website (n = 2)	Patient Society (n = 1)	Total (Average Across All Sources N = 30)	P-value
DISCERN Score	33.07	41.60	44.27	44.27	74.67	39.08	0.07

A detailed ranking of the top five highest-scoring websites according to the DISCERN instrument, alongside their average traffic share, can be found in Appendices D-E.

## Discussion

Traditionally, primary care and specialist physicians have been the principal source of health information for most patients, including but not limited to treatment options. This dynamic is now outdated and has shifted to a shared decision-making approach between patients and physicians, and has rightfully empowered patients to be more actively involved in their own care.

However, there is an ongoing crisis within the UK healthcare system caused by inadequate funding, chronic understaffing, and, more recently, the COVID-19 pandemic, which has led to inflated waiting times and increasing demand for an already stretched health service. This, combined with the advent of the smartphone and easy access to a wealth of information, quite literally at your fingertips, has resulted in a growing number of patients turning to the internet in search of answers to questions regarding their complex health conditions. Furthermore, the impetus towards digitalising medical consultations is contributing to the all-time high usage of the internet, with UK adults spending on average one full day online every week [26].

There is a vast array of online information sources, many of which have not been written or at least validated by healthcare professionals or experts in the field. When combined with the poor literacy level of an average Briton [14], there is a rapid spread of inaccurate information. Not only does this jeopardise a patient-doctor relationship due to conflicting opinions, but it also risks patient safety as some practices are potentially detrimental to one’s health.

An even greater challenge is posed when complex conditions such as AERD are cited. These diagnoses are evasive even for general physicians and often require specialist input for optimal management. Therefore, it would seem apt that most of the websites identified in this study were designed by specialists in the field (46.7%) offering their services. However, the overall quality of the information they provide was, on average, ‘poor’ (DISCERN = 33.07); thus, patients cannot rely on these providers to make an informed decision regarding their treatment options. In addition, only 7.1% (one of the 14 clinical providers) of these providers are UK-based, but all have a shared financial motive, given that the treatment options available are costly and exclusive to the private care sector. This negatively impacts the overall credibility of the health information provided, as there is a degree of implicit bias to market their services. Furthermore, the non-UK websites may offer services that are not recognised by the Medicines and Healthcare products Regulatory Agency (MHRA), as other international boards, such as the Food and Drug Administration (FDA), have differing regulations.

The best-performing clinical provider was Global Allergy & Airway Patient Platform, a ‘fair’ resource with a DISCERN score of 45.87. Although it provides unbiased information, the reading level is equivalent to that of an 18-year-old (FRES = 48.6), and it does not provide any references for where the information was obtained. While we can assume that these were written by experts in the field, this still only corresponds to the lowest level of evidence [27].

As UK-based websites for AERD are limited, it is of great importance that these are published in accordance with Health Education England guidelines and have a readability of the average 11-year-old. However, the sole UK-based clinical provider ("ENT 150 Harley Street") fails to achieve this standard and has a GFI of 12.26, a reading level higher than that expected of the average person. In addition, with a DISCERN score of 29.47, it confirms that this is a poor source of information and requires a great deal of improvement.

Interestingly, the website that achieved the best overall DISCERN score of 74.67, a near-perfect score, is a patient-run advocacy group called Samter’s Society. However, the FRES is still considered to be at the level of an average 17-year-old. Therefore, regardless of the high quality of clinical information provided, poor readability suggests that this information is not easily understood by the average patient, thus contributing to skewed decision-making and a reduced uptake of the appropriate service.

Patient education is paramount for optimal management of health conditions, as it has been shown to improve outcomes and reduce diagnostic delay, which can expedite the initiation of treatment pathways that are best suited to an individual [17]. However, it is evident that high-quality online resources on AERD are scarce, and to ensure that there is an effective dissemination of the correct information, patients, healthcare professionals, and website publishers all have roles to play.

Patients should be encouraged to discuss any information they have retrieved online with a healthcare professional prior to making decisions regarding their care, whilst healthcare professionals can signpost patients to appropriate, unbiased, and evidence-based websites, such as Samter’s Society. Patient information websites and clinical providers should also work collectively to guarantee that their data is presented in accordance with literacy guidelines to allow patients to comprehend complex information in order to educate themselves and make well-informed decisions.

Another suggestion is that all health information websites adopt a similar approach to a relatively new algorithm established by Facebook that identifies and flags misinformation using a combination of feedback from users and the International Fact-Checking Network [28].

This was particularly pertinent during the early stages of the COVID-19 pandemic and has proven to be a useful tool in highlighting and disputing the often dangerous dissemination of incorrect medical advice.

This study is subject to several limitations. First, the methodology restricted the search to the top 40 results across only three search engines, which may exclude relevant patient information found further down the search pages or on alternative search platforms. Furthermore, because Yahoo's search algorithm is largely powered by the Bing index, utilising both as independent engines may have introduced substantial result overlap prior to duplicate exclusion. Second, the explicit exclusion of multimedia and social media platforms narrows the scope of this study, as it does not capture the increasingly prevalent video and user-generated content that patients frequently access. Third, the dynamic and constantly changing nature of internet algorithms means these search results represent a specific point in time rather than a permanent digital landscape. Furthermore, there is a significant temporal lag between the initial data collection in August 2021 and publication, which is a notable constraint when assessing a rapidly evolving digital information landscape. Between-source comparisons were limited by small and unequal group sizes, including two categories containing only two websites each. These comparisons should therefore be interpreted cautiously. Finally, while readability formulas provide a standardised metric for text complexity, they cannot measure a patient's prior medical knowledge, nor do they account for website layout and visual aids, all of which significantly impact a user's true comprehension.

## Conclusions

The online information available regarding AERD and treatment options is of overall poor quality and is written to a reading level higher than what is recommended by Health Education England. The issues surrounding readability are compounded by a lack of quality and reliability; thus, the intended purpose of informing patients is not achieved.

Future studies should continue to evaluate the quality of online information sources, and efforts should be made to ensure that these resources are evidence-based and comprehensible by the public. In addition, health information should be validated and quality-controlled before being posted online. Furthermore, social media platforms increasingly appear to serve as a medium for the rapid spread of information. Although there are some beneficial uses, in the context of health, such as public health communication, it also provides a readily available platform for non-experts to potentially spread inaccurate information. Efforts should also be made to regulate and investigate multimedia/social media content to ensure these are clinically accurate.

## Appendices

Appendix A 

**Table 6:** This table presents the calculations used by the Gunning-Fog Index, Flesch-Kincaid Grade Level and Flesch Reading Ease Score As detailed in Table 8, while the exact mathematical coefficients differ, all three tools calculate reading difficulty by analysing two core metrics: average sentence length and word complexity (typically measured by syllables). FRES: Flesch Reading Ease Score; FKGL: Flesch-Kincaid Grade Level; GFI: Gunning-Fog Index References: [22-24]

Readability Tool	Formula
GFI	0.4 x [(average number of words per sentence) + 100 x (average number of 3 syllable words)]
FKGL	0.39 x (average number of words per sentence) + 11.8 x (average number of syllables per word) - 15.59
FRES	206.835 - 1.015 x (average number of words per sentence) - 8.46 x (average number of syllables per word)

Appendix B 

**Table 7:** The Flesch Reading Ease Score (FRES), estimated UK school level and readability level yo: years old; GCSE: General Certificate of Secondary Education.

FRES	School Level	Readability
0-30	Postgraduate/21+yo	Very difficult
30-50	Undergraduate/18-21yo	Difficult
50-60	GCSE-A-level/14-18yo	Fairly difficult
60-70	Year 9/13-14yo	Standard
70-80	Year 8/12-13yo	Fairly easy
80-90	Year 7/11-12yo	Easy
90-100	Year 6/10-11yo	Very easy

Appendix C 

**Table 8:** DISCERN Questionnaire, ranked 1-5 where 1 = no, 3 = Partially and 5 = Yes References: [25]

Section 1: Is the publication reliable?	
1.	Are the aims clear?
2.	Does it achieve its aims?
3.	Is it relevant?
4.	Is it clear what sources of information were used to compile the publication (other than author or producer
5.	Is it clear when the information used or reported in the publication was produced?
6.	Is it balanced and unbiased?
7.	Does it provide details of additional sources of support and information?
8.	Does it refer to areas of uncertainty?
Section 2: How good is the quality of information on treatment choices?	
9.	Does it describe how each treatment works?
10.	Does it describe the benefits of each treatment?
11.	Does it describe the risks of each treatment?
12.	Does it describe what would happen if no treatment is used?
13.	Does it describe how the treatment choices affect overall quality of life?
14.	Is it clear that there may be more than one possible treatment choice?
15.	Does it provide support for shared decision-making?
Section 3: Overall rating of the publication	
16.	Based on the answers to all of the above questions, rate the overall quality of the publication as a source of information about treatment choices

Appendix D 

**Table 9:** Top 5 Websites Ranked by Overall Quality (DISCERN Score) References: [25]

Rank	Website	Average DISCERN Score
1	Samtersociety.org	74.67
= 2	Aerd.partners.org	54.40
= 2	Asthma.net	54.40
= 3	Healthline.com	52.27
= 3	Verywellhealth.com	53.37

Appendix E 

**Table 10:** Top 5 Websites Ranked by Google Search Prominence and Traffic Share AT: Austria; NZ: New Zealand; US : United States; FRES: Flesch Reading Ease Score; FKGL: Flesch-Kincaid Grade Level; GFI: Gunning-Fog Index

Rank	Website	Flesch-Kincaid grade level	Flesch reading ease score	Gunning-fog index	DISCERN tool	Average traffic share
1	Global Allergy & Airway Patient Platform (AT)	10	48.6	13.18	45.87	32.5%
2	Allergy New Zealand (NZ)	11	46	14.33	29.87	17.6%
3	Healthline (US)	10.5	41.9	12.25	52.27	11.4%
4	Samters Society (US)	9.2	45.4	11.61	74.67	8.1%
5	Chacko Allergy, Asthma and Sinus Center (US)	9.9	49	13.44	41.60	6.1%
